# Data Science Methods and Tools for Industry 4.0: A Systematic Literature Review and Taxonomy

**DOI:** 10.3390/s23115010

**Published:** 2023-05-23

**Authors:** Helder Moreira Arruda, Rodrigo Simon Bavaresco, Rafael Kunst, Elvis Fernandes Bugs, Giovani Cheuiche Pesenti, Jorge Luis Victória Barbosa

**Affiliations:** 1 Applied Computing Graduate Program, University of Vale do Rio dos Sinos, 950, Unisinos Av., São Leopoldo 93022-000, RS, Brazil; rsimonb@edu.unisinos.br (R.S.B.); rafaelkunst@unisinos.br (R.K.); 2 HT Micron Semiconductors S.A., 1550, Unisinos Av., São Leopoldo 93022-750, RS, Brazil; elvis.bugs@htmicron.com.br (E.F.B.); giovani.pesenti@htmicron.com.br (G.C.P.)

**Keywords:** Industry 4.0, data science, machine learning, literature review, taxonomy

## Abstract

The Fourth Industrial Revolution, also named Industry 4.0, is leveraging several modern computing fields. Industry 4.0 comprises automated tasks in manufacturing facilities, which generate massive quantities of data through sensors. These data contribute to the interpretation of industrial operations in favor of managerial and technical decision-making. Data science supports this interpretation due to extensive technological artifacts, particularly data processing methods and software tools. In this regard, the present article proposes a systematic literature review of these methods and tools employed in distinct industrial segments, considering an investigation of different time series levels and data quality. The systematic methodology initially approached the filtering of 10,456 articles from five academic databases, 103 being selected for the corpus. Thereby, the study answered three general, two focused, and two statistical research questions to shape the findings. As a result, this research found 16 industrial segments, 168 data science methods, and 95 software tools explored by studies from the literature. Furthermore, the research highlighted the employment of diverse neural network subvariations and missing details in the data composition. Finally, this article organized these results in a taxonomic approach to synthesize a state-of-the-art representation and visualization, favoring future research studies in the field.

## 1. Introduction

A way of better understanding the current civilization is through the industrial revolution timeline. The first phase of this movement began in the late 18th century, based on the evolution of mechanical equipment for manufacturing and the emergence of steam machines. Then, at the beginning of the 20th century, the possibility of implementing large-scale production based on task division started the second phase of the industrial revolution with the advent of electricity. Afterward, in the early 1970s, the usage of electronics associated with information technology enabled the automation of manufacturing processes, establishing the third phase of this movement [1]. Today, the world lives the so-called new wave of the industrial revolution which started in Europe and spread worldwide [2]. The fourth phase of this revolution, named Industry 4.0, employs technological advances and concepts such as the Internet of things (IoT) and cyberphysical systems (CPS) to assist in the development of smart factories [3,4].

Along with the aforesaid advances, the expression “Data Science” began to be discussed by the information technology community in the first decade of the 21st century. Data scientists are people who deal with significant quantities of data from different sources to extract relevant information in decision-making [5]. One of data science’s main goals is to predict outcomes considering the domain knowledge of interest [6]. A successful data scientist must have a perspective of business problems, in addition to the knowledge of data mining algorithms, computational methods, and software tools to extract knowledge and insights from big datasets [7].

Frequently, these datasets organize observations in high dimensionality with various data types, formats, and sizes. In this sense, one of the most frequent ways to deal with this information is in the time domain. Observations sampled in the time domain constitute a sequence of information named time series [8]. Time series may receive diverse processing methods to understand machinery maintenance, production life cycle, and industrial and business processes to generate valuable outcomes for companies. Moreover, time series allow the aggregation, combination, and computational processing of data to create higher information levels, such as contextual data [9]. Context, in turn, features a situation regarding individuals, applications, and the surrounding environment. Contexts represent the time and the state of something that can be an object, a machine, a system, a person, or a group.

In this regard, the literature presents systematic reviews encompassing the aforementioned scope similar to this study. Manufacturing has generated research studies to deal with decision-making problems using analytical techniques, data mining, and machine learning [10]. Moreover, a review of big data tools and applications for manufacturing presented the essential components to create complete solutions [11]. In addition to case studies applied to a chemical company, a review of data mining and analytical categories such as predictive, inquisitive, descriptive, and prescriptive categories focused on manufacturing processes [12]. However, these reviews do not retrieve and analyze data science methods and software tools focused on general industrial applications. This article proposes a systematic literature review of data science methods and tools employed in distinct segments of the industry. Moreover, the study analyses the usage of different time series levels and data quality concerning data science applications. In this sense, the article provides the answers to three general, two focused, and two statistical questions to synthesize the literature through a taxonomy, favoring the findings’ representation.

The remainder of this article has the following structure. Section 2 describes related works and how this study differentiates from them. Section 3 explains the methodology employed in the systematic review. Section 4 presents the results and the findings based on the research questions, highlighting industrial segments, data science methods, and software tools. Section 5 depicts the proposed taxonomy to represent the findings covered by the literature, and Section 6 discusses the findings. Finally, Section 7 approaches the limitations, future work, and conclusions of this study.

## 2. Related Work

This section analyzes surveys and reviews in comparison to the proposed work. Over the last years, some authors have reviewed the literature, aiming to exploit the best techniques used by smart factories that correspond to the data science field. This is because Industry 4.0 allows the employment of multiple types of technologies in different segments of manufacturing.

Mazzei and Ramjattan [13] used natural language processing techniques to review machine learning methods used in Industry 4.0 cases. The authors stated questions regarding Industry 4.0 main problems, which machine learning methods were used in these situations, and how the areas focused on the academic literature and white papers. The systematic review focused on two databases using the topic modeling technique BERTopic. The most recurrent problems regarded security, smart production, IoT connectivity, service optimization, robotic automation, and logistics optimization. Convolutional neural networks were the most frequent machine learning method.

Wolf et al. [10] studied the lack of management tools oriented toward decision-making problems in the manufacturing domain. The work provided a systematic mapping review that identified seven application areas for data analytics and had advanced analytical techniques associated with each area. The mapping originated a novel tool to ease decision-making that identified promising analytic projects. Moreover, the management tool employed data mining techniques and machine learning algorithms.

Cui et al. [11] published a systematic literature review aiming to classify big data tools with similarities and identify the differences among them. The work took into account industrial data, big data technologies, and data applications in manufacturing. The conceptual framework of the systematic literature review had three perspectives: data source, big data ecosystem, and the data consumer. Data types, source devices, data dynamics, data formats, and systems composed the data source perspective. The big data ecosystem perspective presented data aspects as storage, resource management, visualization, analysis, database, data warehouse, search, query, processing, ingestion, data flow, workflow, and management. Prediction, optimization, monitoring, design, decision support, data analytics, scheduling, data management, simulation, and quality control were part of the components of the data consumer perspective. Four research questions featured the drivers and requirements for big data applications, the essential components of the big data ecosystem, the capabilities of big data ecosystems, and the future directions of big data applications. In conclusion, the authors found six key drivers and nine essential components of the big data ecosystem. The study did not find any enterprise-ready big data solution in the literature.

Belhadi et al. [12] systematically reviewed the literature regarding big data analytics in manufacturing processes in addition to multiple case studies applied to a leading chemical company. The three cases were part of a digital transformation project, the first case being an implementation of big data analytics in a fertilizer plant, the second in a phosphoric acid company, and the third one, an intelligent and self-controlled production unit. The article classified the selected works according to data mining and analytics categories: predictive, inquisitive, descriptive, and prescriptive. Moreover, the implemented techniques categorized papers into offline and real-online. Moreover, the work established the following research trends: real-time data mining approaches, big data analytics enabler architecture, integrated human-data intelligence, and prescriptive analytics. Each research trend pointed to the research questions regarding performance management, production control, and maintenance in manufacturing processes. The authors realized that the emergence of advanced technologies, particularly sensors, generated data with a wide variability, large variety, high velocity, intense volatility, high volume, unascertained veracity, and low value. Furthermore, the study proposed a framework of big data analytics in the manufacturing process, which presented the process challenges, faculties, and capabilities of big data analytics.

None of the related works retrieved and analyzed data science methods and software tools focused on industrial applications (Table 1). Therefore, this article identifies and organizes industrial segments, data science methods, and software tools employed in industrial environments to produce a taxonomy. In turn, the taxonomy synthesizes the literature favoring the representation of the findings. For this, the article describes a systematic literature review converging towards three main themes: Industry 4.0, data science, and time series. These themes are the basis to create general, focused, and statistical questions that shape this work’s investigation. In this sense, the study also investigates specific approaches derived from these themes, particularly the usage of context and the data quality employed in studies. These aspects provide the differential approach of this article regarding the aforementioned reviews.

## 3. Methodology

**Figure 1:**
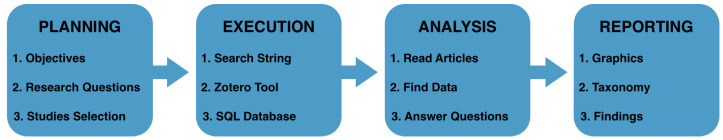
Sequence of the four stages of the research: planning, execution, analysis and reporting. Each stage is organized into three substeps.

This section presents the research methods employed in this work. The structure follows the methodology proposed by Petersen [14]. Figure 1 summarizes the stages organized into four steps with three substeps each. First, the stages encompass the research planning, followed by the execution of the systematic review, analysis of the data, and reporting of the results.

### 3.1. Research Planning

The research planning establishes the objectives, defines the research questions, and plans the selection of the studies. The following subsections explain each step in detail.

#### 3.1.1. Objectives

A systematic review of the state of the art in data science methods and tools employed in Industry 4.0 is the central aspect of this article. The goal was to find studies that employ Industry 4.0, data science, and time series to produce useful insights for the industrial field. After collecting the papers, the objectives concerned the classification of each study according to the industrial segments, data science methods, and software tools. Afterward, this work synthesized the results with graphics, tables, and a taxonomy of the findings to ease the data analysis.

#### 3.1.2. Research Questions

The research questions focused on the three main themes of the review: “Industry 4.0”, “Data Science” and “Time Series”. The seven research questions had the following division: three general questions (GQ), two focused questions (FQ), and two statistical questions (SQ), as shown in Table 2.

The motivation to look for the industrial segments involved with data science was to find out where big quantities of data needed to be analyzed and show new work opportunities (GQ1), the kinds of methods used for this purpose (GQ2), and what were the techniques employed in industry (GQ3). Moreover, understanding how the data are used over time is key to choosing the best technique to use in specific situations (FQ1). Furthermore, the quality of the datasets available is important to analyze how well an algorithm performs related to data gaps and balance (FQ2). Finally, the sources (SQ1) and the number of publications over time (SQ2) help the research process.

#### 3.1.3. Studies Selection

The process of selecting the studies involved five relevant databases in the field of research: ACM, IEEE, Scopus, Springer, and Wiley. A study regarding the research questions helped to define the search string. Moreover, the usage of synonyms and related words allowed the search to get more embracing results. Table 3 shows the organization of the search string considering three themes.

The refining of the search occurred using six exclusion criteria (EC). First, the filtering process disregarded the papers not written in English (EC1) and not found in journals, conferences, or workshops (EC2). Next, the titles (EC3) and abstracts (EC4) analysis only considered the works in agreement with the research questions. Then, the filtering excluded duplicated papers (EC5). Finally, the last filtering criteria (EC6) was the three-pass approach. This approach uses the analysis of the title, abstract, introduction, title of sections and subsections, mathematical content, and conclusions in the first pass. The second pass is the observation of the images, diagrams, and illustrations. At last, the third pass searches the entire text [15].

### 3.2. Execution

After the planning phase, the execution of the planned steps occurred according to the search string’s insertion in the selected databases. Further, the usage of the Zotero tool and an SQL database allowed us to organize the results.

#### 3.2.1. Search String

**Figure 2:**
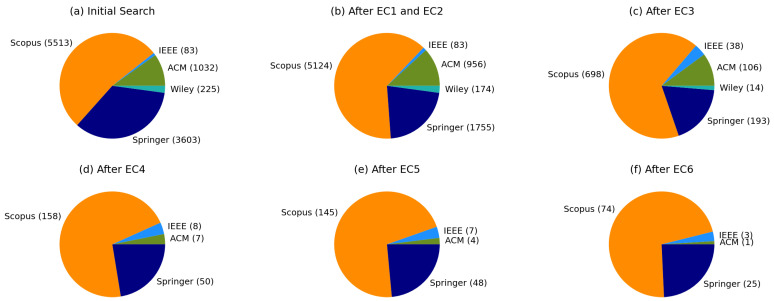
The number of papers retrieved from each database: (**a**) from the initial search; (**b**) after exclusion criteria 1 and 2; (**c**) after exclusion criterion 3; (**d**) after exclusion criterion 4; (**e**) after exclusion criterion 5; (**f**) after exclusion criterion 6. Exclusion criterion 4 discarded the remaining papers from Wiley. Scopus had the greatest number of works selected for the corpus, followed by Springer, IEEE, and ACM.

The databases’ initial search occurred with no filters, using the proposed search string and organizing the data gathered in collections named according to each database. The filtering process occurred all in the “zotero.sqlite” file, which is the SQL database generated by Zotero. The chosen search databases were ACM, IEEE, Scopus, Springer, and Wiley. Figure 2 shows the name of the databases and the number of papers retrieved from the initial search and after applying each exclusion criterion.

#### 3.2.2. Zotero Tool

A single management tool’s usage aims to ease the collecting process, smoothing the papers’ search and classification. A tool with open access to its database is preferable. At the beginning of this study, tests were conducted with the Mendeley (https://www.mendeley.com; accessed on 17 May 2023) and Zotero (https://www.zotero.org; accessed on 17 May 2023) reference management tools. Zotero was chosen, due to the authors’ need of accessing the SQL database with no restrictions, since it is an open-access database. Zotero is a reference manager tool that provides a practical way of gathering papers. It organizes the search results thanks to the possibility of using a browser connector that makes the process faster, by allowing the metadata gathering of a set of papers instead of one by one. Moreover, the use of the ZotFile (http://zotfile.com; accessed on 17 May 2023) browser plugin in the individual analysis of the selected papers eased the extraction of highlighted sentences [16].

Table 4 presents the exclusion criteria used in the filtering process with the Zotero tool. In the main screen of Zotero, the field called “Extra” allows the user to insert additional information about the papers. The appending of the pipe symbol (“|”) to the end of the “Extra” field created a new field to be used by SQL queries called “Status”. This new field used along the filtering process assigned a different “Status” to every paper after applying each exclusion criterion. Before the application of the exclusion criteria, all the papers had the “Status” set to empty (“ ”). The usage of SQL sentences in the Zotero database provided a practical way to apply the first two exclusion criteria at the same time, filtering papers not written in English (EC1) and not found in journals, conferences, or workshops (EC2). The papers that met these exclusion criteria had their “Status” set to “ec”, which meant excluded by EC1 or EC2. The remaining papers with an empty status underwent a filtering by the third exclusion criterion, the title analysis (EC3). The discarded papers had their status changed to “ec3”, and the accepted ones to the next step gained the status “ec3_next”. The filtering process continued with the papers with the status “ec3_next”, which had their abstracts analyzed in the fourth exclusion criterion (EC4), and accepted to the next phase (“ec4_next”) or rejected (“ec4”). The next filter eliminated duplicated works, representing the fifth exclusion criterion (EC5), by setting the status to “ec5” or keeping the paper in the next phase, setting the status to “ec5_next”. The last exclusion criterion (EC6) applied the three-pass approach and changed the status of the discarded papers to “ec6” and of the accepted papers to “final”.

#### 3.2.3. SQL Database

**Figure 3:**
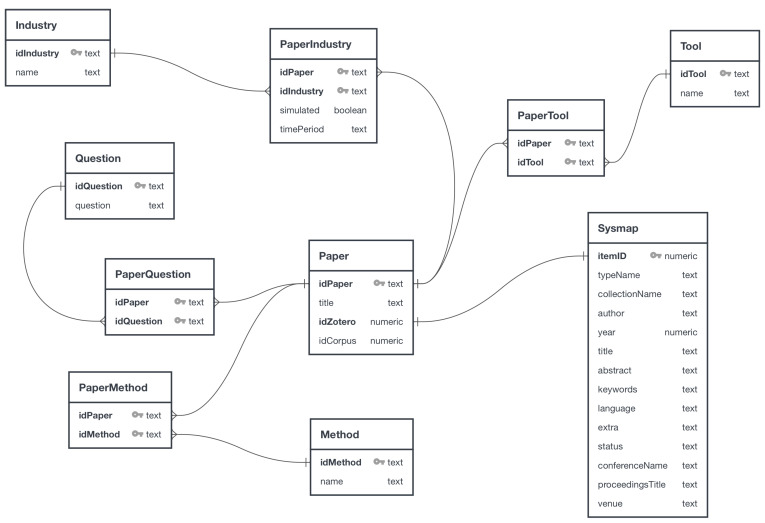
The diagram shows nine tables created to support the systematic review and a view with the essential data of the Zotero database. The table “Paper” is the central entity and has a one-to-one relationship with the view “Sysmap”. The other main tables are “Industry”, “Question”, “Tool”, and “Method”, besides the auxiliary tables “PaperIndustry”, “PaperQuestion”, “PaperTool”, and “PaperMethod”.

The SQL database allowed an organization of the data extracted during the process. Furthermore, the relational model enabled us to organize the data collected over the development of the systematic review and eased the generation of graphics and the extraction of information. Nine tables and a database view of the Zotero tool composed the model. Figure 3 depicts the relational model, developed with the QuickDBD (https://app.quickdatabasediagrams.com; accessed on 17 May 2023) diagram tool.

The table “Paper” had four attributes, a unique identifier of the paper (field “idPaper”), a field to store the title of the work (“title”), an identifier code of the work in the Zotero tool (“idZotero”), and a field with the order of the article in the corpus (“idCorpus”). This table had a one-to-one relationship with the view “Sysmap”, which represented the most relevant data used from the Zotero database.

The field “itemID”, of the view “Sysmap”, was the unique identifier of the paper used by Zotero and it was related to the field “idZotero”, of the table “Paper”. The field “typeName” represented the type of publication (book section, journal article, conference paper, manuscript, book, or report). This work only considered journal articles, conference papers, and workshops, which are a variant of conferences. The field “collectionName” was the name of the collection chosen to organize the documents. This work used the names of the search databases and an identifier representing the search round. The field “author” was the name of the first author. The field “year” was the year of publication, “title” was the title of the article, and “abstract” was the abstract of the paper. The field “keywords” organized the keywords of the work separated by a comma. The “language” was the writing language of the paper. The field “extra” was used to set a status for each paper using a pipe character followed by a code. Another attribute called “status” showed the status code. Papers from a conference or workshop used the fields “conferenceName” and “proceedingsTitle” to store the conference or workshop name and the title of the proceedings. Finally, the field “venue” indicated whether the paper was from a journal, conference, or workshop.

The main tables “Industry”, “Question”, “Tool”, and “Methods” related to the table “Paper” in a disjoint many-to-many relationship into one-to-many relationships with auxiliary tables. The table “Industry” had the register of the industrial segments used in the review. “Question” stored the research questions of the paper. The table “Tool” held the software tools used in the selected papers. The table “Method” had the data science methods implemented by the works. The auxiliary tables “PaperIndustry”, “PaperQuestions”, “PaperTool”, and “PaperMethod” had the primary keys of the main tables. The auxiliary table “PaperIndustry” had two extra fields. One of them was responsible for indicating when a specific industrial segment acted in a simulated environment (field “simulated”) and the other one for storing the time period of the data used in the work (field “timePeriod”).

### 3.3. Analysis

The selected works were carefully investigated looking for data to answer the research questions and classify each work in a specific industry segment. Moreover, the investigation allowed the identification of the data science methods and software tools applied in the studies. Although some papers mentioned the industrial segment, their data actually resulted from a simulation environment. Furthermore, the time duration of data used in the studies, when available, appeared in hours, days, months, or years.

### 3.4. Reporting

The reporting provided results in different ways. The creation of graphics favored the analysis process providing information in figures with data grouped and organized. In addition, the creation of a taxonomy synthesized a general view of the results. Furthermore, the research questions had the answers discussed which produced research highlights.

## 4. Results

**Figure 4:**
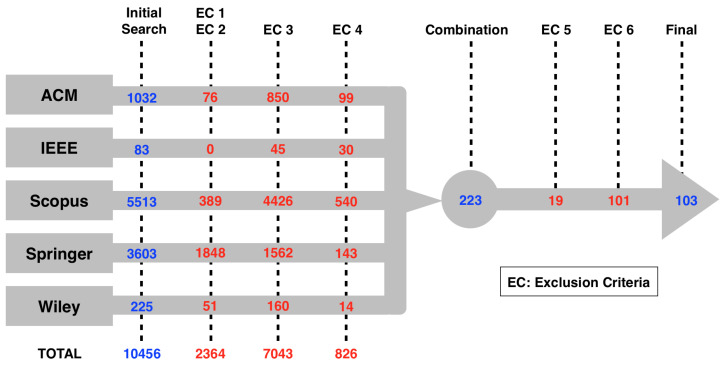
The figure shows the five databases used in the study (ACM, IEEE, Scopus, Springer, and Wiley) with the number of papers discarded after each one of the exclusion criteria applied. The number of papers after the initial search, the combination, and the final step is shown in blue. The number of papers discarded by the exclusion criteria is displayed in red.

This section presents the results of the systematic literature review. Figure 4 shows each step of the process with the number of papers from each database used along the process. Moreover, the figure depicts the number of papers discarded by the exclusion criteria.

First, the initial search returned 10,456 papers from the five databases. With the aim of finding the first years that matched the string, the search did not use any filter besides the keywords present in the search string, which meant no cut by years. Then, the two initial exclusion criteria (EC1 and EC2) removed the papers not written in English and the ones not found in journals, conferences, or workshops (22.61%). The third exclusion criterion (EC3) removed the papers which did not pass the title analysis (67.36%). The fourth exclusion criterion (EC4) excluded papers according to the abstract analysis (7.90%). The combination of the remaining papers resulted in 223 works, representing 2.14% of the initial search. The fifth exclusion criterion (EC5) removed 19 duplicated studies. Finally, the sixth exclusion criteria (EC6) excluded 101 papers using the three-pass approach, leaving 103 works in the corpus, which corresponded to 0.99% of the initial search. Table A1, of Appendix A, shows the selected papers and the corpus identification codes.

The next step consisted of a thorough analysis of the corpus aiming to answer each research question, showing the results with graphics and tables. The rest of this section presents the research questions and respective answers.

### 4.1. GQ1: Which Industrial Segments Applied Data Science Techniques?

Aiming to standardize the industrial segments present in the corpus, these results considered the classification proposed by the International Labour Organization (https://www.ilo.org; accessed on 17 May 2023), a United Nations agency. This classification presents 22 industrial segments, of which 15 were in the corpus. Table 5 shows the industrial segments and each paper’s corpus identification code, besides an extra segment for papers with segments fitted in the general-purpose use segment.

The *general purpose/others* industrial segment represented the major number of papers with 24.04% related to the corpus’s total. After, *mechanical and electrical engineering* was the second industrial segment with 19.23%, followed by *transport equipment manufacturing* with 15.38%. The other segments represented less than 10% of the total each. Luo et al. [17] used two industrial segments: *transport equipment manufacturing* and *Utilities (water, gas, and electricity)*. That paper was accounted twice for percentage analysis purposes.

*Utilities* represented 8.65% of the corpus. *basic metal production* approached 6.73% of the corpus. *Oil and gas* represented 5.77% of the corpus. *Health services* and *mining* encompassed 3.85% each. *Food* represented 2.88% of the corpus. *Agriculture*, *postal and telecommunications services*, and *textiles* encompassed 1.92% of the corpus each. *Chemical industries*, *construction*, *forestry*, and *media* approached 0.96% of the corpus each.

### 4.2. GQ2: What Are the Data Science Methods Used in the Studies?

A primordial aspect of the successful use of data science is the choice of suitable methods. Table 6 shows the abbreviations of the data science methods used in each paper, ordered by the corpus identification code, and Table A2 of Appendix B contains the names of the methods. Long short-term memory (LSTM) was the most used data science method, appearing in 22 papers, followed by support vector machine (SVM), with 19 appearances, and random forest (RF), which appeared 14 times. Convolutional neural network (CNN) appeared 11 times. Recurrent neural network (RNN) appeared nine times. Multilayer perceptron (MLP) and Principal component analysis (PCA) appeared eight times each. Neural network (NN) appeared seven times. Autoregressive integrated moving average (ARIMA) and logistic regression (LR) appeared six times each. Autoencoder (AE), deep neural network (DNN), local outlier factor (LOF), and synthetic minority oversampling technique (SMOTE) appeared five times each. Convolutional neural network–long short-term memory (CNN-LSTM), density-based spatial clustering of applications with noise (DBSCAN), gated recurrent unit (GRU), K-means (KM), K-nearest neighbor (KNN), one-class SVM (OCSVM), support vector regression (SVR), and XGBoost (XGB) appeared four times each. AdaBoost (AB), bidirectional long short-term memory (BLSTM), backpropagation neural network (BPNN), decision tree (DT), gradient boosting decision tree (GBDT), Gaussian mixture models (GMM), hidden Markov models (HMM), linear regression model (LRM), and isolation forest (iForest) appeared three times each. Agglomerative hierarchical clustering (AHC), attention-based long short-term memory (ALSTM), artificial neural network (ANN), bidirectional gated recurrent unit (BGRU), Bayesian ridge/regularization (BR), classification and regression tree (CART), fault detection and classification convolutional neural network (FDC-CNN), gradient boosting machine (GBM), hierarchical clustering algorithm/analysis (HCA), linear discriminant analysis (LDA), matrix profile (MP), ontology (Ontology), self-organizing maps (SOM), short-term Fourier transform (STFT), visual analytics (VA), and wide-first kernel and deep convolutional neural network (WDCNN) appeared two times each. The other data science methods appeared just one time each over the corpus.

**Figure 5:**
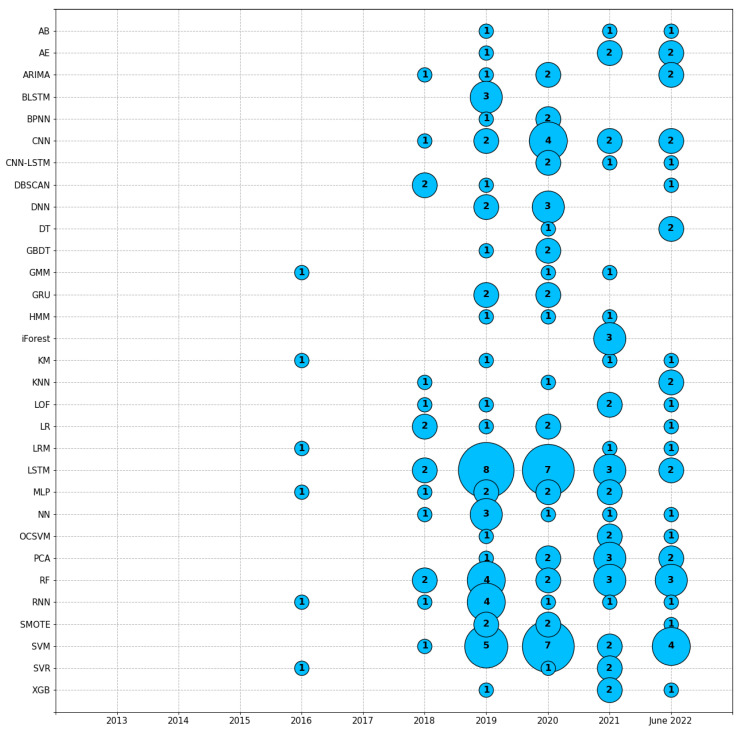
Data science methods grouped by year. The definition of each method is in Table A2. Long short-term memory—LSTM was the method with the most occurrences (22), followed by support vector machine—SVM (19), and random forest—RF (14). For better visualization, only methods with more than two occurrences appear in the picture.

Furthermore, to better follow the evolution over the timeline, Figure 5 shows how many times a data science method appeared over the years of publication. Long short-term memory (LSTM) networks were the method that most appeared in the corpus, with 22 occurrences. Then, support vector machine (SVM) had 19 occurrences. Next, the random forest (RF) method appeared 14 times. The years 2019, 2020, and 2021 presented the highest concentration of data science methods.

### 4.3. GQ3: What Are the Software Tools Used in the Studies?

Implementing data science methods requires proper software tools such as programming languages, databases, and toolkits. Table 7 shows the abbreviation of the software tools used in each paper of the corpus, and Table A3 of Appendix C, contains the complete names of the tools. Python was the most used software tool, appearing in 20 papers, followed by Keras, in 15 papers, and Tensorflow in 13. MATLAB appeared in eight works and the R language appeared in six. Hadoop and SKLEARN appeared in five studies each. Kafka and MongoDB appeared in four papers each. Spark appeared in three studies. doParallel, fastcluster, foreach, InfluxDB, JavaScript, Jupyter, Knime, MES, MSSQL, PyTorch, rpud, SQL, Storm, and SWRL appeared in two papers each. The remaining software tools appeared just once in the corpus.

**Figure 6:**
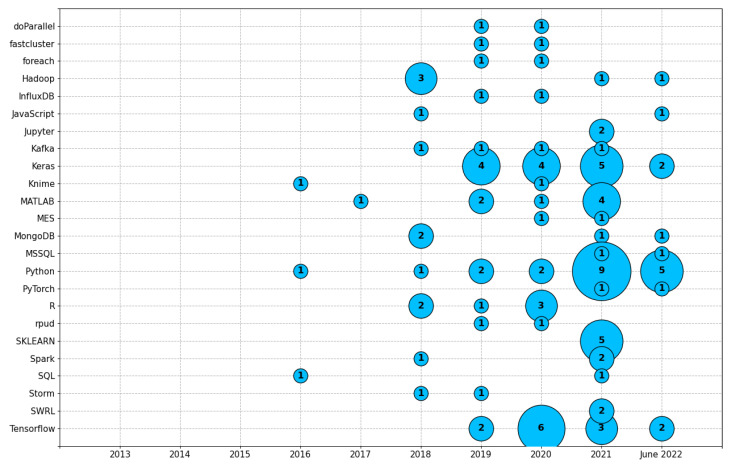
Software tools grouped by year. The definition of each tool is in Table A3. Python was the tool with the most occurrences (20), followed by Keras (15), and Tensorflow (13). For a better visualization, only tools with more than one occurrence appear in the picture.

Moreover, Figure 6 shows the software tools grouped by years. The Python programming language was the most used tool, appearing in 20 papers, followed by Keras, which appeared in 15 papers, and Tensorflow which appeared in 13 articles.

### 4.4. FQ1: How Do the Studies Employ Contextual Time Series?

Eleven papers used the concept of context in some way. The works approached ontologies, visual analytics, dynamic Bayesian networks, context-aware cyberphysical systems, convolutional neural networks, recurrent neural networks, and long short-term memory networks.

Wu et al. [18] used context information to develop an interactive visual analytics system for a petrochemical plant. The system worked in the operation stage, using time-series data from 791 sensors which provided the status of different parts of the factory. Tripathi and Baruah et al. [19] proposed a method to identify contextual anomalies in a time-series-modifying dynamic Bayesian network (DBN) method to support context information, named contextual DBN. The tests of the new method efficacy occurred in oil well drilling data. Majdani et al. [20] developed a framework for cyberphysical systems using machine learning and computational intelligence. The framework used context data from 25 sensors of different parts of a gas turbine. Canizo et al. [21] proposed a convolutional neural network–recurrent neural network (CNN-RNN) architecture to extract features and learn the temporal patterns of context-specific time-series data from 20 sensors installed at a service elevator.

Jiang et al. [22] used two deep learning methods to predict the remaining useful life (RUL) of bearings. The methods employed context vectors in time-series multiple-channel networks for convolutional neural networks (TSMC-CNN) and extended the method to attention-based long short-term memory networks (TSMC-CNN-ALSTM). Stahl et al. [23] presented a case of steel sheets’ failure detection using bidirectional recurrent neural networks (RNN) with an attention mechanism. The method used context vectors to represent each state of the process. Ma et al. [24] proposed a predictive production planning architecture based on big data for a ceramic manufacturing company. The architecture used cube-based models to deal with context-aware historical data using LSTM networks. Yasaei et al. [25] developed an adaptive context-aware and data-driven model using measures from 62 heterogeneous sensors of a wastewater plant. The model used LSTM networks to detect sensing device anomalies and environmental anomalies.

Abbasi et al. [26] developed an ontology for aquaponic systems called AquaONT, using the methontology approach to formulate and evaluate the model. The ontology used contextual data from a standard farm to provide information on the optimal operation of IoT devices. Bagozi et al. [27] proposed an approach focused on resilient cyberphysical production systems (R-CPPS), exploiting big data and the human-in-the-loop perspective. The study used context-aware data stream partitioning, processing data streams collected in the same context, which means the same smart machine and the same type of process to produce the same kind of product. Kim et al. [28] conducted an experiment to observe the participants’ attentiveness in a repeated workplace hazard, using virtual reality to avoid the risk of injuries. The experiment used a construction task to measure the participants’ biosignals by means of eye-tracking sensors and a wearable device to measure the electrodermal activity, together with contextual features.

### 4.5. FQ2: What Is the Data Quality over Time Used in the Studies?

Data quality is primordial for all types of industrial segments, including the assembly lines of industries. Knowing the quantity of data over time used in an experiment is fundamental for a better understanding of the data analysis. Out of one hundred and three papers in the corpus, the equivalent of 39.81% (41 papers) mentioned the quantity of data used over a certain period of time. Table 8 presents this information along with the paper identification. Despite mentioning the quantity of data, the units of measure appeared in different forms. The years represent the quantity of data in 14 studies, months in 17 works, days express data in 7 papers, and hours in 3 works.

Another crucial point regarding data quality is the origin of the datasets used in the experiments. Table 9 shows ten papers of the corpus that made their datasets available to public. Three papers used the same repository, although two of them focused on Turbofan engine degradation (Lu et al. [29] and Wu et al. [30]), and the other one on bearings (Ding et al. [31]). Shenfield et al. [32] and Kancharla et al. [33], which worked with two datasets, also used bearings but from different repositories. Moreover, Apiletti et al. [34] used data from hard-drives, Mohsen et al. [35] worked on a human activity dataset, Zvirblis et al. [36] used data from conveyor belts, Wahid et al. [37] worked with a component failure dataset, and Zhan et al. [38] used data from wind turbines.

### 4.6. SQ1: In Which Databases Are the Studies Published?

**Figure 7:**
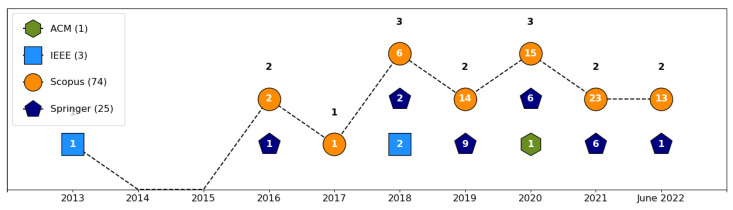
The number of papers in each database by year. Of the five databases used in this work, only four had papers in the corpus. Scopus was the database with the greatest number of studies (74), followed by Springer (25), IEEE (3), and ACM (1). Wiley stayed out of the corpus with no papers selected.

The review applied the searches to five databases: ACM, IEEE, Scopus, Springer, and Wiley. However, only four databases had studies selected into the corpus, as shown in Figure 7. Scopus had the great majority of papers (71.84%), followed by Springer (24.27%), IEEE (2.91%), and ACM (0.97%).

### 4.7. SQ2: What Is the Number of Publications per Year?

**Figure 8:**
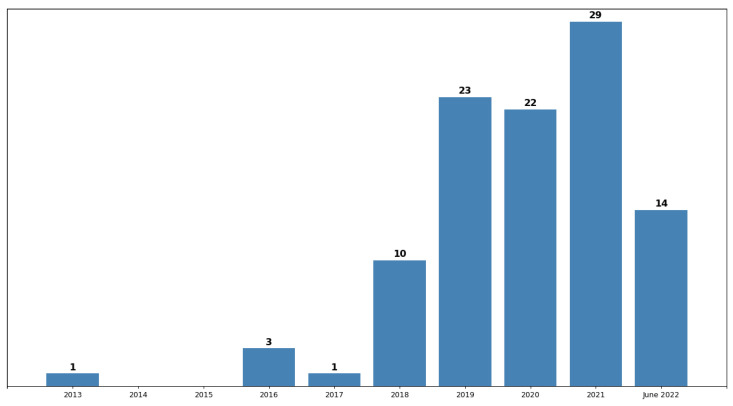
The number of publications present in corpus per year. The years with the higher number of works published were 2019, 2020, and 2021 with 23, 22, and 29 papers, respectively. The years refer to the papers’ publication date.

Over the last five years, the publications related to this study increased, doubling from 2018 (10 papers) to 2019 (23 papers). Figure 8 shows the annual progress of the publications, taking into account the date of publishing. The first publication that fit the selection criteria was in 2013 and the last in 2022. Only fourteen works emerged until the end of June 2022 because this was the date when the searches were executed.

**Figure 9:**
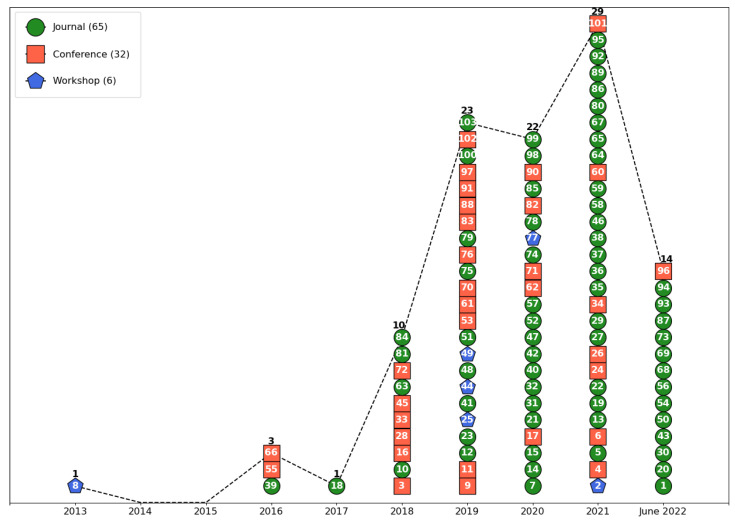
Types of publication by year, classified as conference, journal, or workshop. The number inside the geometric shapes is the identification code of the paper in the corpus. The years 2019, 2020, and 2021 with 23, 22, and 29 papers, respectively, had the biggest number of publications. Overall, there were 65 publications from journals, 32 from conferences, and 6 presented in workshops.

Regarding the types of publications, Figure 9 shows the paper identification code inside a geometric shape. Conference works use a square symbol, journal papers use a circle, and workshop papers use a diamond symbol. Journals had the greatest number of papers (63.11%), followed by conferences (31.07%) and workshops (5.83%).

## 5. Taxonomy

**Figure 10:**
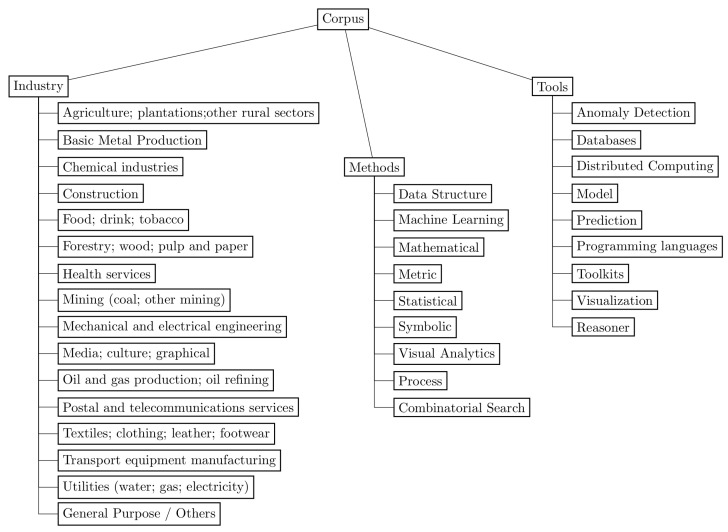
The taxonomy has three main branches: industry, methods, and tools. Industry organizes the papers into industrial segments, according to the International Labour Organization. Methods depict the data science methods employed in the papers. Tools organize the software tools used in the works.

This section summarizes the answers to the three general research questions, previously presented in Table 2, using a taxonomic approach to better visualize and understand the results. Figure 10 depicts a taxonomy that hierarchically organizes, classifies, and synthesizes the industrial segments (GQ1), data science methods (GQ2), and software tools (GQ3) found in the corpus with the nodes *industry* [39], *methods* [40,41,42], and *tools* [43,44], respectively. Industrial segments featured sixteen classes, data science methods organized algorithms and techniques into nine branches, and software tools presented applications and libraries organized into nine components.

The industrial segments used in this work originated from the International Labour Organization (ILO) (https://www.ilo.org/global/industries-and-sectors; accessed on 17 May 2023), an agency of the United Nations, which classifies industries and sectors into 22 segments. The 103 papers resulted from the systematic review fell into 15 of the 22 segments proposed by the ILO: *agriculture*, *basic metal production*, *chemical industries*, *construction*, *food*, *forestry*, *health services*, *mining*, *mechanical and electrical engineering*, *media*, *oil and gas*, *postal and telecommunications services*, *textiles*, *transport equipment manufacturing*, and *utilities*. These different segments complement those industries with *general purpose*.

**Figure 11:**
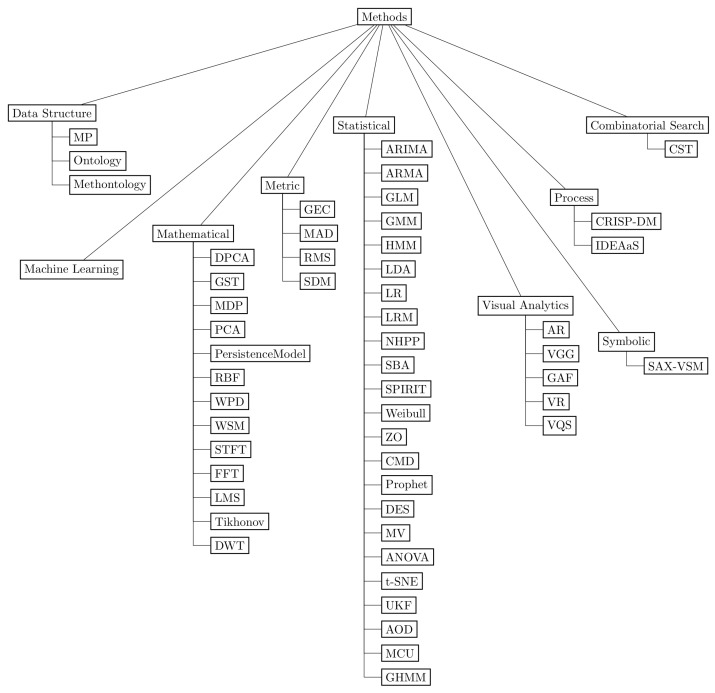
The methods branch presents the data science methods split into data structure, machine learning, mathematical, metric, statistical, symbolic, visual analytics, process, and combinatorial search. As a result of the significant number of specialized methods, the machine learning branch is presented in more detail in Figure 12.

**Figure 12:**
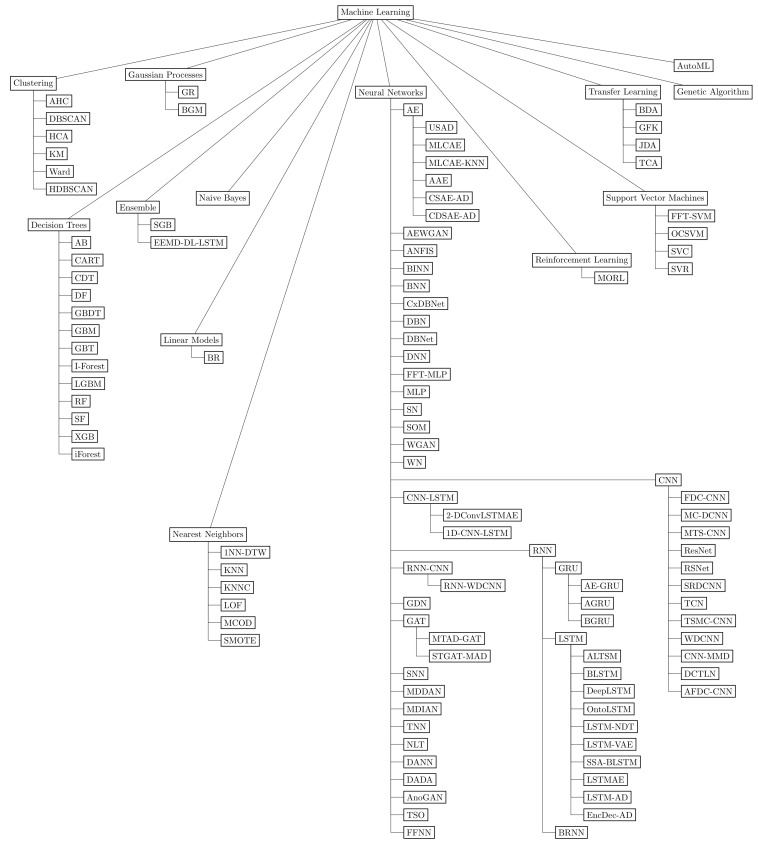
Machine learning branch has the following organization: clustering, decision trees, ensemble, Gaussian processes, linear models, naive Bayes, nearest neighbors, neural networks, reinforcement learning, support vector machines, transfer learning, genetic algorithm, and AutoML.

The data science methods found included *data structure*, *machine learning*, *mathematical*, *metric*, *statistical*, *symbolic*, *visual analytics*, *process*, and *combinatorial search*, as shown in the taxonomy and more detailed in Figure 11. Due to the significant number of methods and their variations, the *machine learning* branch had a separated taxonomy shown in Figure 12. The machine learning method *long short-term memory* (LSTM) networks represented the most used method, with 22 occurrences. Furthermore, there were ten LSTM variations: *attention-based long short-term memory* (ALSTM), which uses a context vector to infer different attention degrees of distinct data features at specific time points [22]; *bidirectional long short-term memory* (BLSTM), which processes data both in chronological order, from start to end, and in the opposite direction, the reverse order [21,23]; *deep long short-term memory* (DeepLSTM), an LSTM network with stacked layers connected to a dense layer distributed over time [45]; *long short-term memory with nonparametric dynamic thresholding* (LSTM-NDT) [38]; *long short-term memory variational autoencoder* (LSTM-VAE) [38]; *singular spectrum analysis bidirectional long short-term memory* (SSA-BLSTM) [46]; *long short-term memory autoencoder* (LSTMAE) [47]; *long short-term memory anomaly detection* (LSTM-AD) [48]. *encoder–decoder anomaly detection* (EncDec-AD) [48]; and the *ontology-based LSTM neural network* (OntoLSTM), which implements semantics concepts using an ontology to learn the representation of a production line, together with an LSTM network for temporal dependencies learning [49].

The second most used data science method was the *support vector machine* (SVM) method, representing 19 occurrences. Moreover, the method had four variations: *fast Fourier transform based support vector machines* (FFT-SVM), a version of SVM which uses a fast Fourier transform to extract features [32]; *one-class SVM* (OCSVM), an unsupervised version of SVM using a single class to identify similar or different data [50]; *support vector classification* (SVC), a variation used for classification tasks [34]; and the *support vector regression* (SVR) variation, which implements a linear regression function to the mapped data [51].

The data science method that was the third-most used was the decision tree method *random forest* (RF), accumulating 14 occurrences, followed by *convolutional neural network* (CNN), with 11 occurrences, and *recurrent neural network* (RNN), with 9 occurrences. Twelve CNN variations stood out as branches: *fault detection and classification convolutional neural network* (FDC-CNN), designed to detect multivariate sensor signals’ faults over a time axis, extracting fault features; *multichannel deep convolutional neural networks* (MC-DCNN), whose objective is to deal with multiple sensors that generate data with different lengths; *multiple-time-series convolution neural network* (MTS-CNN), designed for diagnosis and fault detection of time series, uses a multichannel CNN to extract important data features [52]; *temporal convolutional network* (TCN), which works by summarizing signals in time steps, using a maximum and minimum value per step [53]; *residual neural networks* (ResNet) [54]; *residual-squeeze Net* (RSNet) [45]; *stacked residual dilated convolutional neural network* (SRDCNN) [32]; *wide first kernel and deep convolutional neural network* (WDCNN) [32,55]; *convolutional neural network maximum mean discrepancy* (CNN-MMD) [33]; *deep convolutional transfer learning network* (DCTLN) [55]; *attention fault detection and classification convolutional neural network* (AFDC-CNN) [48]; and the *time-series multiple-channel convolutional neural network* (TSMC-CNN), which uses as inputs N-variate time series split into segments, smoothing the extraction of data points [22]. RNN represented three branches: *gated recurrent unit* (GRU), *long short-term memory* (LSTM), and *bidirectional recurrent neural network* (BRNN).

**Figure 13:**
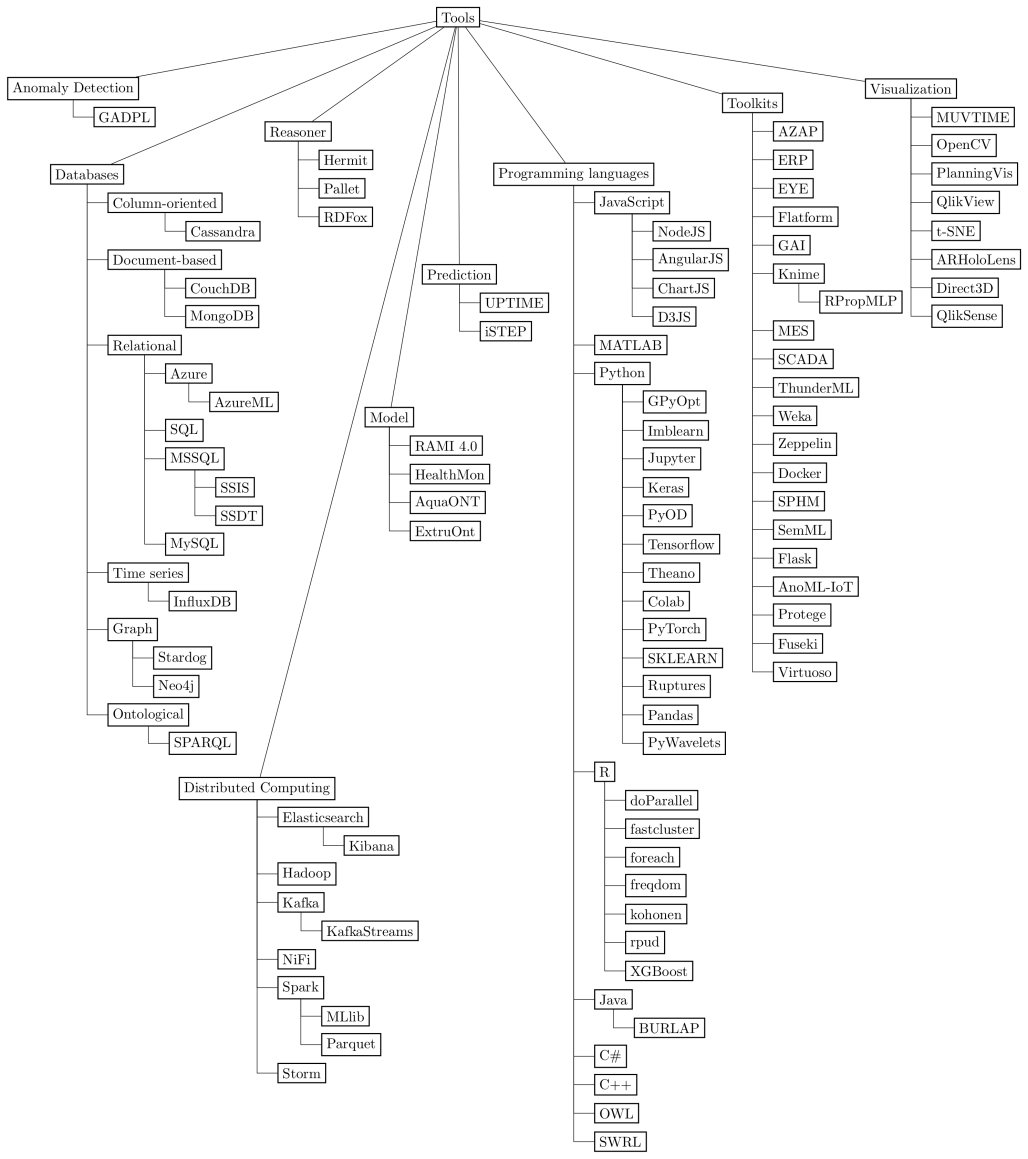
The tools branch presents the software tools used by the authors, split into anomaly detection, databases, distributed computing, model, prediction, programming languages, toolkits, visualization, and reasoner. All the branches represent one or more ramifications.

Regarding the software tools, nine main classes appeared in the taxonomy: *anomaly detection*, *databases*, *distributed computing*, *model*, *prediction*, *programming languages*, *toolkits*, *visualization*, and *reasoner*, as depicted in Figure 13. The *Python* language was the most used software tool, with 20 occurrences, followed by *Keras* (15 occurrences), and *Tensorflow* (13 toccurrences). *Keras* is a deep learning framework, and *Tensorflow* is a machine learning back end [32], and both are branches of *Python* in the taxonomy hierarchy.

Despite covering industrial segments, data science methods, and software tools hierarchically, the taxonomy did not link them horizontally. These relations are in Table 5, representing industrial segments, Table 6 showing data science methods, and Table 7 providing software tools.

## 6. Discussion

The results presented in this study originated from a systematic review process focused on Industry 4.0, data science and time series. There was no restriction regarding the publication year to provide a whole spectrum of literature in these aforementioned fields. With this, the review showed industrial segment applications both from real cases and simulated environments, in addition to identifying data science methods, software tools, and the data quality used by the experiments.

Several industrial segments are interested in analyzing data, and more and more data analysis is crucial for companies. This contributes to decision-making in the function of historical data generated by each industry. Moreover, these data analytical processes contribute to the companies’ specific needs since previous experiences are substantial to improve future outcomes.

The industrial segments explored by the literature were classified and grouped according to the International Labour Organization pattern. This provided a better way of visualization in the taxonomy (Figure 10). The *general purpose/others* industrial segment appeared in 25 papers, being the most present in the corpus. The *mechanical and electrical engineering* industrial segment was the second most common one (20 papers). The segment includes industries strictly connected to technology, such as semiconductors, computers, and electronics, which explains why it was the most frequent segment in the study, after *general purpose/others*. Furthermore, this industry usually has controlled environments and employees trained to work with technology, making the collection of data simpler. This favors the execution of studies because those industrial environments are already prepared to produce data combinations toward high-level decision-making.

The majority of studies used real industrial facilities in the experiments (81 papers). However, some papers employed simulated environments (23 works). The work of Luo et al. [17] appeared twice in the simulated cases due to the presence of two industrial segments in the paper. The usage of real data in most papers provides evidence of the evolution of data science applications in the industry’s production line. This is because sensors and database tools have evolved and become more affordable in the last years. Moreover, the quality of real datasets is a positive point for the training of machine learning algorithms since it can improve the accuracy of predictive models and substantiate future applications that use the same type of data. This is also positive because it reflects real industrial scenarios and potentially provides technology for real-world problems.

Furthermore, the literature presents a wide usage of different technologies, which can hinder the right choice of a suitable method since there is a chance of empirically employing the methods. Aside from the methods, choosing the right tool is another challenge due to different implementations of the same method in distinct tools, e.g., programming languages which present alternative values to initialize the weights of a neural network. A couple of tools rely on specific methods, such as the Keras tool, which deals with deep learning applications employing LSTM and GRU methods. Moreover, it is common to see Keras and Tensorflow tools used together [21,32,54,56,57,58]. Both Keras and Tensorflow support the Python language, which is widely used for scientific purposes, appearing in 20 papers of the corpus, as presented in Table 7. On the other hand, regarding the usage of data combination to create high-level information, the corpus included 11 papers that mentioned contextual data [18,19,20,21,22,23,24,25,26,27,28].

In addition to the aforesaid technologies, neural networks were among the 13 variations of machine learning methods according to the taxonomy. On the other hand, neural networks themselves presented 31 subvariations. With this machine learning method’s improvement, three approaches stood out: attention-based, bidirectional, and autoencoder networks. The attention-based mechanism acts like the human visual attention behavior, using a context vector and focusing on the importance of different features over distinct time steps to improve the prediction accuracy. The studies which focused on this attention-based mechanism explored the usage of, for example, ALSTM and AGRU. Bidirectional models work as two different neural networks walking through a data sequence in both directions to avoid forgotten data. One network goes from the start to the end of the sequence, and the other one comes from the opposite direction. In this respect, studies encompassed the usage of BLSTM, BGRU, and BRNN. An autoencoder is an unsupervised feed-forward neural network commonly used for feature extraction and dimensionality reduction, composed of an encoder and a decoder. The encoder compresses the data to a hidden layer, and the decoder reassembles it to the original input data. In particular, studies used 2-DConvLSTMAE, AEWGAN, AE-GRU, and AE. Hence, these techniques focused on novel combinations and variations of neural networks, which provide versatile methods to exploit problems and questions within the scope of data science in industries.

More specifically, the data quality analysis is critical to ensure a proper functioning of the above-mentioned data science methods. Missing details in the data composition can hamper the paper’s understanding and the reproducibility of the experiment. The quantity of data over time is not enough to supply all the information needed since the frequency can vary during the same period. For example, it is possible to measure the air temperature every hour or every minute of the day. If the measurement occurs every hour, it results in 24 rows. On the other hand, if the measurement occurs every minute, it results in 1440 rows. Therefore, these measurements provide different data granularity, which consequently affects the way results are described. More importantly, these cases require an adequate exposure to methodologies and discussions considering the method’s specificity.

Regarding data structures found in the methods, ontologies provide an advanced way to retrieve information. Classes and relations organize data as a taxonomy but with the possibility to query and reason. The SPARQL is the language used to retrieve information and Hermit, Pallet, and RDFox are examples of reasoners found in the review. An important aspect of ontologies is that they are extendable and reusable [26,49,59].

In addition, another crucial piece of information that studies should clearly provide is the percentage of data used for training and testing the model because this strategy of data splitting directly affects the results. Moreover, to guarantee the experiment’s reproducibility, some specific details of the methods are of significant importance, for example, the number of hidden layers of a neural network, or the type of kernel used by a support vector machine, or even the number of interactions used by a random forest. In this sense, there is a need for studies to present more about the data organization and how the data science methods were employed. Papers must include all details of the implementation, such as the architecture and parameters of the machine learning methods and the whole composition of feature vectors. With this, the practitioners will find the methodologies clearer to understand and reproduce in their studies. Hence, this will benefit the community, ensuring potential common situations among different segments to avoid technical and managerial aspects.

## 7. Conclusions

This article presented a systematic literature review focused on Industry 4.0, data science, and time series. This work investigated the usage of data science methods and software tools in several industrial segments, taking into account the implementation of time series and the data quality employed by the authors. Furthermore, a taxonomy organized the industrial segments, data science methods, and software tools in a hierarchical and synthesized way, which eased the reading of how studies from Industry 4.0 have employed these technologies.

The literature presented several mature methods which covered vast possibilities for industrial analysis. This strengthens both the market and academia because the more companies employ the technologies, the more researchers and practitioners become experts in those methods and tools. In this sense, the industrial investment in these analyses is beneficial because it provides empirical results for the community about applicable use cases in several segments. Moreover, it contributes to the maturity and evolution of the technological methods and tools employed in the process of industrial data analysis.

Even with efforts to reduce biases, this review has limitations as any other systematic review. The search string was applied to five research databases intending to use different academic sources, which potentially decreased the source bias. The search string’s conception used three axes employing respective known keywords and synonyms for each axis, focusing on reducing keywords biases. Moreover, six exclusion criteria filtered the resulting papers, providing the corpus. Accordingly, these exclusion criteria and the remaining filtering process followed Petersen et al.’s [14] guidelines to reduce process bias.

The taxonomy represents an important contribution to further research since the organization of data science methods and software tools helps the visual search in categories, assisting in discovering research gaps. In addition, the variation of a specific method or tool into a node points to trends in the use of that technology, which is important when choosing what technique to use. Therefore, the taxonomy’s faculty of organizing and classifying the results in hierarchical classes constitutes a relevant achievement of this work. Moreover, the class industry was an attempt to standardize the segments according to the International Labour Organization. Hence, the visualization of the outcomes in the form of a taxonomy increases the possibilities of new research.

Finally, this research study did not focus on how the works dealt with data treatment before applying data science methods to datasets. This situation constitutes an additional limitation, and hence, it is suggested as future work. Moreover, how the software tools are linked to the data science methods is another potential future work. Furthermore, the last topic suggested for future work is to specifically correlate the most used methods and tools with each industrial segment.

## Figures and Tables

**Table 1:** Related works and the presence of data science methods and tools compared to this work.

Paper	Methods	Tools
Mazzei and Ramjattan (2022) [13]	Yes	No
Wolf et al. (2019) [10]	No	Yes
Cui et al. (2020) [11]	No	Yes
Belhadi et al. (2019) [12]	Yes	No
This work	Yes	Yes

**Table 2:** The research questions divided into general questions (GQ), focused questions (FQ), and statistical questions (SQ).

Ref.	Research Questions
GQ1	Which industrial segments applied data science techniques?
GQ2	What are the data science methods used in the studies?
GQ3	What are the software tools used in the studies?
FQ1	How do the studies employ contextual time series?
FQ2	What is the data quality over time used in the studies?
SQ1	In which databases are the studies published?
SQ2	What is the number of publications per year?

**Table 3:** The search string and its three themes: “Industry 4.0”, “Data Science” and “Time Series”.

Theme	Search Terms
Industry 4.0	( “industry 4.0” OR “industrie 4.0” OR “cyber physical systems” )
	AND
Data science	( “data science” OR “machine learning” OR “big data”
	OR “data analytics” OR “data mining” )
	AND
Time series	( “time series” OR “context histories” OR “contexts histories”
	OR “context history” OR “trails” )

**Table 4:** Exclusion criteria and status filters used during the corpus selection.

Short	Exclusion Criteria	Status	Excluded	Next Criteria
EC1	Not written in English	“ ”	“ec”	-
EC2	Not found in journals, conferences	“ ”	“ec”	-
	or workshops			
EC3	Title analysis	“ ”	“ec3”	“ec3_next”
EC4	Abstract’s analysis	“ec3_next”	“ec4”	“ec4_next”
EC5	Duplicated papers	“ec4_next”	“ec5”	“ec5_next”
EC6	Three-pass approach	“ec5_next”	“ec6”	“final”

**Table 5:** Industrial segments and the identification codes of the papers in the corpus.

Industrial Segment	Corpus ID
Agriculture, plantations, other rural sectors	35, 59
Basic metal production	13, 14, 19, 25, 50, 66, 103
Chemical industries	89
Construction	86
Food, drink, tobacco	22, 46, 88
Forestry, wood, pulp and paper	53
Health services	9, 18, 49, 58
Mechanical and electrical engineering	4, 11, 12, 16, 20, 26, 45, 52, 56, 61, 62, 72, 76, 78, 79, 80, 82, 87, 90, 102
Media, culture, graphical	100
Mining (coal, other mining)	23, 54, 70, 91
Oil and gas production; oil refining	3, 15, 27, 74, 75, 81
Postal and telecommunications services	63, 85
Textiles, clothing, leather, footwear	48, 51
Transport equipment manufacturing	5, 6, 10, 31, 34, 40, 41, 44, 55, 57, 60, 68, 84, 92, 98, 101
Utilities (water, gas, electricity)	8, 28, 33, 39, 41, 47, 71, 93, 96
General purpose/others	1, 2, 7, 17, 21, 24, 29, 30, 32, 36, 37, 38, 42, 43, 64, 65, 67, 69, 73, 77, 83, 94, 95, 97, 99

**Table 6:** Identification codes of the papers at the corpus and the data science methods used by each one.

ID	Method(s)	ID	Method(s)
1	CNN, GAF	52	BDA, CNN, DT, GFK, JDA, KNN, LDA, SVM, TCA
2	DWT, LRM, NN, STFT	53	MLP
3	RMS	54	ARIMA, DES
4	AFDC-CNN, CDSAE-AD, CSAE-AD, EncDec-AD, FDC-CNN, LSTM-AD	55	LRM, MLP
5	ANFIS, MLP, NHPP, RBF, SVR, Weibull	56	RNN
6	AE, LOF, RF, iForest	57	CNN
7	DPCA, GBDT	58	ANN, SVM
9	RF	59	EEMD-DL-LSTM
10	ARIMA	61	LSTM, OntoLSTM
11	BINN, I-Forest, OCSVM, PCA	62	MCOD, MP
12	BPNN, DBN, DNN, KNNC, SVM, WPD	63	LOF
14	2-DConvLSTMAE, ARIMA, CNN-LSTM, DeepLSTM, PersistenceModel, RSNet	65	CST, GA, KM
15	ARIMA, CNN, LSTM, ResNet	66	RNN, SOM
16	LSTM, RNN, SBA	67	CNN, CNN-LSTM, LSTM
17	GBM, RF, SVM, TCN	68	CRISP-DM, DT, KNN, LRM, Prophet, RF, SVM
18	BR	69	LR, LSTM, RF, SVM, TNN
19	GHMM, HMM, MCU	70	CART, GST, LDA, SDM, SVM
20	AE, VGG	71	CNN-LSTM, LSTM
21	AGRU, ALSTM, FFT-MLP, FFT-SVM, GRU, LSTM, RNN-WDCNN, SRDCNN, WDCNN	72	GBT, LR, RF, SVC
22	AOD	73	AE, CMD, CNN, CNN-MMD, KNN, MDDAN, MDIAN, MLCAE, MLCAE-KNN, SVM
23	AE, BGRU, BLSTM, BRNN, GRU, LSTM, RNN	74	MLP, SMOTE, SVM
24	AML, FFNN, RF, XGB	75	AB, CART, GBDT, LGBM, NN, RF, XGB
25	HMM, LSTM, MDP	76	AHC
26	AE, LOF, TSO, iForest	77	LSTM, MORL
27	VQS	78	AHC, SOM, Ward
28	VA	79	BGRU, BLSTM, CNN, GRU, LSTM, RNN
29	AnoGAN, FFT, LMS, LSTM, OCSVM, PCA, Tikhonov, UKF, t-SNE	80	1NN-DTW, FDC-CNN, MC-DCNN, MTS-CNN, SAX-VSM, SF
30	PCA, SSA-BLSTM	81	CDT, DBSCAN, GEC, KNN, NN
31	AE-GRU, DNN, GRU, LSTM, MLP, RNN	82	AEWGAN, LR, RF, SMOTE, SVM, WGAN
32	CNN, PCA, SVM	83	HCA, KM
33	CNN, LSTM	84	DBSCAN, LR, MLP, NB, RF
35	Methontology	85	WSM
36	BGM, GMM, HDBSCAN, MP, PCA	86	ANOVA, SVM, VR
37	CNN, OCSVM, RNN, iForest	87	CNN-LSTM
38	LSTM	88	LSTM
39	GMM, KM, SPIRIT, SVR	89	AB, GBM, MLP, PCA, RF, SVR, XGB
40	GMM, LSTM	90	DF, LR, NN, SVM
41	BNN, GLM, NN, SGB, SVM	91	HCA
42	ARMA, BPNN, LSTM, SVR	92	Ontology
43	ARIMA, DBSCAN, KM, LOF, LSTM, MV, OCSVM	93	SNN
44	NN	94	AR
45	SVM	96	GDN, LSTM-NDT, LSTM-VAE, MTAD-GAT, STGAT-MAD, USAD
46	IDEAaS	97	ARIMA, CNN, DNN, LSTM, MLP, RF, SN, WN, ZO
47	CxDBNet, DBNet	98	DNN, HMM, PCA
48	ANN, SMOTE	99	ALSTM, BPNN, BR, DNN, GBDT, GR, SVM, TSMC-CNN
49	DBSCAN, LOF, LSTM, MAD, RNN, SMOTE, SVM	100	LSTM, RNN
50	AB, DT, NN, PCA, RF, SMOTE, SVM, XGB	101	Ontology
51	VA	103	BLSTM, LR, RF, SVM

**Table 7:** Identification codes of the papers in the corpus and the software tools used by each one.

ID	Tool(s)	ID	Tool(s)
2	Python, PyWavelets	56	Python, PyTorch
4	Keras, Python, SKLEARN, Tensorflow	57	OpenCV
5	MATLAB	60	Elasticsearch, Flatform, Hadoop, Jupyter, Kafka, Kibana, NiFi, Parquet, Python, Spark, Zeppelin
6	Keras, Python, SKLEARN, Spark, Tensorflow	61	Imblearn
7	CouchDB, freqdom, QlikView, R, XGBoost	63	Cassandra, EYE, Hadoop, R, Spark
10	R	65	HealthMon, MATLAB
11	PyOD	66	SQL
13	Keras, Pandas, Python	67	Jupyter, Python, SKLEARN
14	Python, Tensorflow	68	Hadoop, MySQL, Python
15	GAI, GPyOpt, Keras, Tensorflow	69	Colab
18	MATLAB	70	MATLAB
20	Keras, Tensorflow	72	iSTEP, MLlib
21	Keras, Tensorflow	73	Python
23	Keras	74	Knime, RPropMLP
25	InfluxDB, Kafka, RAMI4.0, Storm, UPTIME	76	doParallel, fastcluster, foreach, R, rpud
27	ExtruOnt, Neo4j, RDFox, SPARQL, Stardog, SWRL, Virtuoso	77	BURLAP, ERP, Kafka, Keras, MES, Tensorflow
28	Hadoop, MongoDB	78	doParallel, fastcluster, foreach, kohonen, R, rpud
29	PyTorch, SKLEARN	79	Keras, Tensorflow
32	Python, R	81	Hadoop
34	MATLAB, MES, MSSQL, QlikSense, SSDT, SSIS	84	JavaScript, Kafka, MongoDB, Python, Storm
35	AquaONT, Fuseki, Hermit, OWL, Pallet, Protege, SWRL	85	PlanningVis
37	AnoML-IoT, Python	86	Ruptures
38	Keras, Python, Tensorflow	87	Keras, Tensorflow
39	Python	88	Azure
42	MATLAB	89	Flask, Keras, Python, SKLEARN
43	AngularJS, ChartJS, D3JS, Docker, JavaScript, MongoDB, NodeJS, Python	90	AzureML
46	MongoDB	92	SemML
47	SCADA	94	ARHoloLens, C#, C++, Direct3D, MSSQL
49	MATLAB	95	MATLAB, SPHM
51	MUVTIME	97	Keras, Python, Tensorflow, ThunderML
52	t-SNE, Tensorflow	98	InfluxDB, KafkaStreams, Keras, Tensorflow
53	AZAP	101	SQL
54	Python	102	GADPL
55	Knime, Weka	103	Keras, Python, Theano

**Table 8:** Quantity of data over time employed in each paper as described by the authors, identified by the ID of the paper in the corpus. The quantity of data appears in years, months, days, and hours.

ID	Quantity	ID	Quantity	ID	Quantity
6	2 days	36	1 year	71	8 days
7	61 days	37	2 days	72	3 years
8	7 days	38	2 years and 6 months	74	4 years and 5 months
10	3655 h	39	1 year	76	2 years
11	5 months	42	1 month	77	3 years
13	3 months	44	1 year	78	6 months
14	1 year	45	1 year	84	8 months
15	4 months	46	1 year	85	30 days
16	2 years	51	8 months	87	1 year
19	3 months	53	7 years	88	242 days
27	1 year	55	3 months	98	50 h
28	3 months	59	2 months	102	7 years
33	3 months	66	50 h	103	6 months
34	6 months	68	1 year and 7 months		

**Table 9:** The papers whose datasets are available to the public, identified by the ID of the paper in the corpus, the author, and the URL where the data can be downloaded. Ten papers presented the dataset used. Accessed on 17 May 2023.

ID	Author	URL
21	Shenfield and Howarth et al. [32]	https://engineering.case.edu/bearingdatacenter/download-data-file
23	Ding et al. [31]	https://ti.arc.nasa.gov/tech/dash/groups/pcoe/prognostic-data-repository/#bearing
31	Lu et al. [29]	https://ti.arc.nasa.gov/tech/dash/groups/pcoe/prognostic-data-repository/#turbofan
40	Wu et al. [30]	https://ti.arc.nasa.gov/tech/dash/groups/pcoe/prognostic-data-repository/#turbofan
67	Mohsen et al. [35]	https://www.kaggle.com/datasets/drsaeedmohsen/wisdmdataset2021
69	Zvirblis et al. [36]	https://github.com/TadasZvirblis/CORBEL
72	Apiletti et al. [34]	https://www.backblaze.com/b2/hard-drive-test-data.html
73	Kancharla et al. [33]	https://engineering.case.edu/bearingdatacenter/download-data-file
		https://mb.uni-paderborn.de/kat/forschung/datacenter/bearing-datacenter
87	Wahid et al. [37]	https://github.com/ashishpatel26/Predictive_Maintenance_using_Machine-Learning_Microsoft_Casestudy
96	Zhan et al. [38]	https://github.com/zhanjun717/STGAT

## Appendix A. Corpus

**Table A1:** Corpus of articles derived from this research.

ID	Author	Title	Venue
1	Toma et al., (2022) [60]	A Bearing Fault Classification Framework Based on Image Encoding Techniques and a Convolutional Neural Network under Different Operating Conditions	Journal
2	Onus et al., (2021) [61]	A Case Study on Challenges of Applying Machine Learning for Predictive Drill Bit Sharpness Estimation	Workshop
3	Rezende et al., (2018) [62]	A case study on the analysis of an injection moulding machine energy data sets for improving energy and production management	Conference
4	Tchatchoua et al., (2021) [48]	A Comparative Evaluation of Deep Learning Anomaly Detection Techniques on Semiconductor Multivariate Time Series Data	Conference
5	Soltanali et al., (2021) [63]	A comparative study of statistical and soft computing techniques for reliability prediction of automotive manufacturing	Journal
6	Ribeiro et al., (2021) [64]	A Comparison of Anomaly Detection Methods for Industrial Screw Tightening	Conference
7	Zhang et al., (2020) [65]	A CPPS based on GBDT for predicting failure events in milling	Journal
8	Ding et al., (2013) [66]	A Data Analytic Engine Towards Self-Management of Cyber-Physical Systems	Workshop
9	Mulrennan et al., (2019) [67]	A data science approach to modelling a manufacturing facility’s electrical energy profile from plant production data	Conference
10	Subramaniyan et al., (2018) [68]	A data-driven algorithm to predict throughput bottlenecks in a production system based on active periods of the machines	Journal
11	Carletti et al., (2019) [50]	A deep learning approach for anomaly detection with industrial time series data: A refrigerators manufacturing case study	Conference
12	Li et al., (2019) [69]	A deep learning driven method for fault classification and degradation assessment in mechanical equipment	Journal
13	Bampoula et al., (2021) [47]	A Deep Learning Model for Predictive Maintenance in Cyber-Physical Production Systems Using LSTM Autoencoders	Journal
14	Essien and Giannetti et al., (2020) [45]	A Deep Learning Model for Smart Manufacturing Using Convolutional LSTM Neural Network Autoencoders	Journal
15	Villalobos et al., (2020) [54]	A flexible alarm prediction system for smart manufacturing scenarios following a forecaster–analyzer approach	Journal
16	Fu et al., (2018) [70]	A Hybrid Forecasting Framework with Neural Network and Time-Series Method for Intermittent Demand in Semiconductor Supply Chain	Conference
17	Van Herreweghe et al., (2020) [53]	A Machine Learning-Based Approach for Predicting Tool Wear in Industrial Milling Processes	Conference
18	Alexopoulos and Packianather et al., (2017) [71]	A monitoring and data analysis system to achieve zero-defects manufacturing in highly regulated industries	Journal
19	Sarda et al., (2021) [72]	A Multi-Step Anomaly Detection Strategy Based on Robust Distances for the Steel Industry	Journal
20	Cordoni et al., (2022) [73]	A multi–modal unsupervised fault detection system based on power signals and thermal imaging via deep AutoEncoder neural network	Journal
21	Shenfield and Howarth et al., (2020) [32]	A novel deep learning model for the detection and identification of rolling element-bearing faults	Journal
22	da Silva Arantes et al., (2021) [74]	A novel unsupervised method for anomaly detection in time series based on statistical features for industrial predictive maintenance	Journal
23	Ding et al., (2019) [31]	A predictive maintenance method for shearer key parts based on qualitative and quantitative analysis of monitoring data	Journal
24	Zufle et al., (2021) [75]	A Predictive Maintenance Methodology: Predicting the Time-to-Failure of Machines in Industry 4.0	Conference
25	Bousdekis et al., (2019) [76]	A RAMI 4.0 View of Predictive Maintenance: Software Architecture, Platform and Case Study in Steel Industry	Workshop
26	Tedesco et al., (2021) [77]	A Scalable Deep Learning-Based Approach for Anomaly Detection in Semiconductor Manufacturing	Conference
27	Berges et al., (2021) [78]	A Semantic Approach for Big Data Exploration in Industry 4.0	Journal
28	Wu et al., (2018) [18]	A Visual Analytics Approach for Equipment Condition Monitoring in Smart Factories of Process Industry	Conference
29	Tagawa et al., (2021) [79]	Acoustic Anomaly Detection of Mechanical Failures in Noisy Real-Life Factory Environments	Journal
30	Mahmood et al., (2022) [46]	An accurate detection of tool wear type in drilling process by applying PCA and one-hot encoding to SSA-BLSTM model	Journal
31	Lu et al., (2020) [29]	An autoencoder gated recurrent unit for remaining useful life prediction	Journal
32	Kiangala and Wang et al., (2020) [80]	An Effective Predictive Maintenance Framework for Conveyor Motors Using Dual Time-Series Imaging and Convolutional Neural Network in an Industry 4.0 Environment	Journal
33	Yue et al., (2018) [81]	An End-to-End model based on CNN-LSTM for Industrial Fault Diagnosis and Prognosis	Conference
34	Vicencio et al., (2021) [82]	An Intelligent Predictive Maintenance Approach Based on End-of-Line Test Logfiles in the Automotive Industry	Conference
35	Abbasi et al., (2021) [26]	An ontology model to represent aquaponics 4.0 system’s knowledge	Journal
36	Nieves Avendano et al., (2021) [83]	Anomaly detection and event mining in cold forming manufacturing processes	Journal
37	Kayan et al., (2021) [84]	AnoML-IoT: An end to end re-configurable multi-protocol anomaly detection pipeline for Internet of Things	Journal
38	Mateus et al., (2021) [85]	Anticipating Future Behavior of an Industrial Press Using LSTM Networks	Journal
39	Vries et al., (2016) [51]	Application of machine learning techniques to predict anomalies in water supply networks	Journal
40	Wu et al., (2020) [30]	Approach for fault prognosis using recurrent neural network	Journal
41	Luo et al., (2019) [17]	Big data analytics–enabled cyber-physical system: model and applications	Journal
42	Ma et al., (2020) [24]	Big data driven predictive production planning for energy-intensive manufacturing industries	Journal
43	Rousopoulou et al., (2022) [86]	Cognitive analytics platform with AI solutions for anomaly detection	Journal
44	Hoppenstedt et al., (2019) [87]	CONSENSORS: A Neural Network Framework for Sensor Data Analysis	Workshop
45	Chen et al., (2018) [88]	Construct an Intelligent Yield Alert and Diagnostic Analysis System via Data Analysis: Empirical Study of a Semiconductor Foundry	Conference
46	Bagozi et al., (2021) [27]	Context-Based Resilience in Cyber-Physical Production System	Journal
47	Tripathi and Baruah et al., (2020) [19]	Contextual Anomaly Detection in Time Series Using Dynamic Bayesian Network	Journal
48	Park et al., (2019) [89]	Cyber Physical Energy System for Saving Energy of the Dyeing Process with Industrial Internet of Things and Manufacturing Big Data	Journal
49	Rousopoulou et al., (2019) [90]	Data Analytics Towards Predictive Maintenance for Industrial Ovens	Workshop
50	Kim and Lee et al., (2022) [91]	Data-analytics-based factory operation strategies for die-casting quality enhancement	Journal
51	Varela et al., (2019) [92]	Decision support visualization approach in textile manufacturing a case study from operational control in textile industry	Journal
52	Azamfar et al., (2020) [93]	Deep Learning-Based Domain Adaptation Method for Fault Diagnosis in Semiconductor Manufacturing	Journal
53	Bibaud-Alves et al., (2019) [94]	Demand forecasting using artificial neuronal networks and time series: Application to a French furniture manufacturer case study	Conference
54	Wang et al., (2022) [95]	Design of PM2.5 monitoring and forecasting system for opencast coal mine road based on internet of things and ARIMA Mode	Journal
55	Majdani et al., (2016) [20]	Designing a Context-Aware Cyber Physical System for Smart Conditional Monitoring of Platform Equipment	Conference
56	Wang et al., (2022) [96]	Detecting anomalies in time series data from a manufacturing system using recurrent neural networks	Journal
57	El Wahab et al., (2020) [97]	Detection and Control System for Automotive Products Applications by Artificial Vision Using Deep Learning	Journal
58	Garmaroodi et al., (2021) [98]	Detection of Anomalies in Industrial IoT Systems by Data Mining: Study of CHRIST Osmotron Water Purification System	Journal
59	Eze et al., (2021) [99]	Developing a Novel Water Quality Prediction Model for a South African Aquaculture Farm	Journal
60	Akin et al. (2021) [100]	Enabling Big Data Analytics at Manufacturing Fields of Farplas Automotive	Conference
61	Huang et al., (2019) [49]	Enhancing deep learning with semantics: An application to manufacturing time series analysis	Conference
62	Naskos et al., (2020) [101]	Event-Based Predictive Maintenance on Top of Sensor Data in a Real Industry 4.0 Case Study	Conference
63	Kurpanik et al., (2018) [102]	EYE: Big data system supporting preventive and predictive maintenance of robotic production lines	Journal
64	Jang and Cho et al., (2021) [55]	Feature Space Transformation for Fault Diagnosis of Rotating Machinery under Different Working Conditions	Journal
65	de Lima et al., (2021) [103]	HealthMon: An approach for monitoring machines degradation using time-series decomposition, clustering, and metaheuristics	Journal
66	Zurita et al., (2016) [104]	Industrial process monitoring by means of recurrent neural networks and Self Organizing Maps	Conference
67	Mohsen et al., (2021) [35]	Industry 4.0-Oriented Deep Learning Models for Human Activity Recognition	Journal
68	Mosavi et al., (2022) [105]	Intelligent energy management using data mining techniques at Bosch Car Multimedia Portugal facilities	Journal
69	Zvirblis et al., (2022) [36]	Investigation of deep learning models on identification of minimum signal length for precise classification of conveyor rubber belt loads	Journal
70	Ghosh and Banerjee et al., (2019) [106]	IoT-based seismic hazard detection in coal mines using grey systems theory	Conference
71	Yasaei et al., (2020) [25]	IoT-CAD: context-aware adaptive anomaly detection in IoT systems through sensor association	Conference
72	Apiletti et al., (2018) [34]	iSTEP, an Integrated Self-Tuning Engine for Predictive Maintenance in Industry 4.0	Conference
73	Kancharla et al., (2022) [33]	Latent Dimensions of Auto-Encoder as Robust Features for Inter-Conditional Bearing Fault Diagnosis	Journal
74	Orru et al., (2020) [107]	Machine learning approach using MLP and SVM algorithms for the fault prediction of a centrifugal pump in the oil and gas industry	Journal
75	Min et al., (2019) [108]	Machine Learning based Digital Twin Framework for Production Optimization in Petrochemical Industry	Journal
76	Kovács and Ko et al., (2019) [109]	Machine Learning Based Monitoring of the Pneumatic Actuators’ Behavior Through Signal Processing Using Real-World Data Set	Conference
77	Lepenioti et al., (2020) [56]	Machine Learning for Predictive and Prescriptive Analytics of Operational Data in Smart Manufacturing	Workshop
78	Kovacs and Ko et al., (2020) [110]	Monitoring Pneumatic Actuators’ Behavior Using Real-World Data Set	Journal
79	Canizo et al., (2019) [21]	Multi-head CNN–RNN for multi-time series anomaly detection: An industrial case study	Journal
80	Hsu and Liu et al., (2021) [52]	Multiple time-series convolutional neural network for fault detection and diagnosis and empirical study in semiconductor manufacturing	Journal
81	Khodabakhsh et al., (2018) [111]	Multivariate Sensor Data Analysis for Oil Refineries and Multi-mode Identification of System Behavior in Real-time	Journal
82	Song and Baek et al., (2020) [112]	New anomaly detection in semiconductor manufacturing process using oversampling method	Conference
83	Ooi et al., (2019) [113]	Operation status tracking for legacy manufacturing systems via vibration analysis	Conference
84	Syafrudin et al., (2018) [114]	Performance analysis of IoT-based sensor, big data processing, and machine learning model for real-time monitoring system in automotive manufacturing	Journal
85	Sun et al., (2020) [115]	PlanningVis: A Visual Analytics Approach to Production Planning in Smart Factories	Journal
86	Kim et al., (2021) [28]	Predicting workers’ inattentiveness to struck-by hazards by monitoring biosignals during a construction task: A virtual reality experiment	Journal
87	Wahid et al., (2022) [37]	Prediction of Machine Failure in Industry 4.0: A Hybrid CNN-LSTM Framework	Journal
88	Sonthited et al., (2019) [116]	Prediction of production performance for tapioca industry using LSTM neural network	Conference
89	Ayvaz and Alpay et al., (2021) [117]	Predictive maintenance system for production lines in manufacturing: A machine learning approach using IoT data in real-time	Journal
90	Quatrini et al., (2020) [118]	Predictive model for the degradation state of a hydraulic system with dimensionality reduction	Conference
91	Brzychczy and Trzcionkowska et al., (2019) [119]	Process-Oriented Approach for Analysis of Sensor Data from Longwall Monitoring System	Conference
92	Zhou et al., (2021) [120]	SemML: Facilitating development of ML models for condition monitoring with semantics	Journal
93	Baquerizo et al., (2022) [121]	Siamese Neural Networks for Damage Detection and Diagnosis of Jacket-Type Offshore Wind Turbine Platforms	Journal
94	Becher et al., (2022) [122]	Situated Visual Analysis and Live Monitoring for Manufacturing	Journal
95	Sundaram and Zeid et al., (2021) [123]	Smart Prognostics and Health Management (SPHM) in Smart Manufacturing: An Interoperable Framework	Journal
96	Zhan et al., (2022) [38]	Stgat-Mad: Spatial-Temporal Graph Attention Network For Multivariate Time Series Anomaly Detection	Conference
97	Shrivastava et al., (2019) [57]	ThunderML: A Toolkit for Enabling AI/ML Models on Cloud for Industry 4.0	Conference
98	Chen et al., (2020) [58]	Time Series Data for Equipment Reliability Analysis with Deep Learning	Journal
99	Jiang et al., (2020) [22]	Time series multiple channel convolutional neural network with attention-based long short-term memory for predicting bearing remaining useful life	Journal
100	Rehse et al., (2019) [124]	Towards Explainable Process Predictions for Industry 4.0 in the DFKI-Smart-Lego-Factory	Journal
101	Zhou et al., (2021) [59]	Towards Ontology Reshaping for KG Generation with User-in-the-Loop: Applied to Bosch Welding	Conference
102	Gras et al., (2019) [125]	Unsupervised Anomaly Detection in Production Lines	Conference
103	Stahl et al., (2019) [23]	Using recurrent neural networks with attention for detecting problematic slab shapes in steel rolling	Journal

## Appendix B. Methods

**Table A2:** Methods.

Method	Name
1D-CNN-LSTM	One-dimensional convolutional neural network long short-term memory
1NN-DTW	One-nearest-neighbor with dynamic time warping
2-DConvLSTMAE	Deep convolutional LSTM stacked autoencoder for univariate, multistep machine speed forecasting
AAE	Attentional autoencoder
AB	AdaBoost
AE	Autoencoder
AE-GRU	Autoencoder gated recurrent unit
AEWGAN	Autoencoder Wasserstein generative adversarial networks
AFDC-CNN	Attention fault detection and classification convolutional neural network
AGRU	Attention-based gated recurrent unit
AHC	Agglomerative hierarchical clustering
ALSTM	Attention-based long short-term memory
AML	AutoML
ANFIS	Adaptive neuro-fuzzy inference system
ANN	Artificial neural network
AnoGAN	Anomaly detection generative adversarial networks
ANOVA	Analysis of variance
AOD	Anomaly and outlier detector
AR	Augmented reality
ARIMA	Autoregressive integrated moving average
ARMA	Autoregressive moving average
BDA	Balanced distribution adaptation
BGM	Bayesian Gaussian mixture
BGRU	Bidirectional gated recurrent unit
BINN	Bayesianly interpretable neural network
BLSTM	Bidirectional long short-term memory
BNN	Bayesian neural network
BPNN	Back propagation neural network
BR	Bayesian ridge/regularization
BRNN	Bidirectional recurrent neural network
CART	Classification and regression tree
CDSAE-AD	Convolutional denoising sparse autoencoders anomaly detection
CDT	Complex decision tree
CMD	Central mean discrepancy
CNN	Convolutional neural network
CNN-LSTM	Convolutional neural network–long short-term memory
CNN-MMD	Convolutional neural network maximum mean discrepancy
CRISP-DM	Cross-industry standard process for data mining
CSAE-AD	Convolutional sparse autoencoders anomaly detection
CST	Combinatorial search of two
CxDBNet	Contextual dynamic Bayesian network
DADA	Discriminative adversarial domain adaptation
DANN	Domain-adversarial training of neural networks
DBN	Deep belief network
DBNet	Dynamic Bayesian network
DBSCAN	Density-based spatial clustering of applications with noise
DCTLN	Deep convolutional transfer learning network
DeepLSTM	Deep long short-term memory
DES	Double exponential smoothing method
DF	Decision forest
DNN	Deep neural network
DPCA	Dynamic principal component analysis
DT	Decision tree
DWT	Discrete wavelet transformation
EEMD-DL-LSTM	Ensemble empirical mode decomposition and deep learning long short-term memory
EncDec-AD	Encoder–decoder anomaly detection
FDC-CNN	Fault detection and classification convolutional neural network
FFNN	Feed-forward neural network
FFT	Fast Fourier transformation
FFT-MLP	Fast Fourier transform based multilayer perceptron
FFT-SVM	Fast Fourier transform based support vector machines
GA	Genetic algorithm
GAF	Gramian angular field
GBDT	Gradient boosting decision tree
GBM	Gradient boosting machine
GBT	Gradient-boosted tree
GDN	Graph deviation network
GEC	Gross error classification
GFK	Geodesic flow kernel
GHMM	Gaussian hidden Markov models
GLM	Generalized linear model
GMM	Gaussian mixture models
GR	Gaussian regression
GRU	Gated recurrent unit
GST	Grey systems theory
HCA	Hierarchical clustering algorithm/analysis
HDBSCAN	Hierarchical density-based spatial clustering of applications with noise
HMM	Hidden Markov models
I-Forest	Isolation forest
IDEAaS	Interactive data exploration as-a-service
iForest	Isolation forest
JDA	Joint distribution adaptation
KM	K-means
KNN	K-nearest neighbors
KNNC	K-nearest-neighbor classification
LDA	Linear discriminant analysis
LGBM	LightGBM
LMS	logMelSpectrogram
LOF	Local outlier factor
LR	Logistic regression
LRM	Linear regression model
LSTM	Long short-term memory
LSTM-AD	Long short-term memory anomaly detection
LSTM-NDT	LSTM with nonparametric dynamic thresholding
LSTM-VAE	Long short-term memory variational autoencoder
LSTMAE	LSTM-Autoencoder
MAD	Mean absolute deviation
MC-DCNN	Multichannel deep convolutional neural networks
MCOD	Streaming distance-based outlier detection algorithm
MCU	Minimum covariance determinant
MDDAN	Multiscale deep domain-adaptive network
MDIAN	Multiscale deep intraclass adaptive network
MDP	Markov decision process
Methontology	Methontology
MLCAE	Multilayer convolutional autoencoder
MLCAE-KNN	Multilayer convolutional autoencoder K-nearest neighbors
MLP	Multilayer perceptron
MORL	Multiobjective reinforcement learning
MP	Matrix profile
MTAD-GAT	Multivariate time-series anomaly detection via graph attention network
MTS-CNN	Multiple time-series convolution neural network
MV	Majority voting
NB	Naive Bayes
NHPP	Nonhomogeneous Poisson process
NLT	Neural linear transformation
NN	Neural network
OCSVM	One-class SVM
Ontology	Ontology
OntoLSTM	Ontology-based LSTM neural network
PCA	Principal component analysis
PersistenceModel	Operates on the assumption that the predicted value remains unchanged from the previous time lag
Prophet	Prophet
RBF	Radial basis function
ResNet	Residual neural networks
RF	Random forest
RMS	Root mean square
RNN	Recurrent neural network
RNN-WDCNN	Recurrent neural network with a wide first kernel and deep convolutional neural network
RSNet	Residual-squeeze net
SAX-VSM	Symbolic aggregate approximation and vector space model
SBA	Syntetos–Boylan Approximation
SDM	Seismic detection method
SF	Shapelet forests
SGB	Stochastic gradient boosting
SMOTE	Synthetic minority oversampling technique
SN	SeriesNet
SNN	Siamese neural networks
SOM	Self-organizing maps
SPIRIT	Streaming pattern discovery on multiple time series
SRDCNN	Stacked residual dilated convolutional neural network
SSA-BLSTM	Singular spectrum analysis bidirectional long short-term memory
STFT	Short-term Fourier transform
STGAT-MAD	Spatial–temporal graph attention network for multivariate time series anomaly detection
SVC	Support vector classification
SVM	Support vector machine
SVR	Support vector regression
t-SNE	t-Distributed stochastic neighbor embedding
TCA	Transfer component analysis
TCN	Temporal convolutional network
Tikhonov	Tikhonov
TNN	Transformer neural network
TSMC-CNN	Time-series multiple-channel convolutional neural network
TSO	Tournament search optimization
UKF	Unscented Kalman filter
USAD	Unsupervised anomaly detection for multivariate time series
A	Visual analytics
VGG	Visual geometry group
VQS	Visual query system
VR	Virtual reality
Ward	Wards method
WDCNN	Wide-first kernel and deep convolutional neural network
Weibull	Weibull Model
WGAN	Wasserstein generative adversarial networks
WN	WaveNet
WPD	Wavelet packet decomposition
WSM	Weighted sum model
XGB	XGBoost
ZO	Zero order

## Appendix C. Tools

**Table A3:** Tools.

Tool	Name
AngularJS	AngularJS
AnoML-IoT	AnoML-IoT
AquaONT	AquaONT
ARHoloLens	AR HoloLens
AZAP	Software suite
Azure	Database
AzureML	Azure Machine Learning Studio
BURLAP	Brown-UMBC Reinforcement Learning and Planning library
C#	Programming language
C++	Programming language
Cassandra	Database
ChartJS	ChartJS
Colab	Google Colaboratory Platform
CouchDB	Amazon CouchDB
D3JS	D3JS
Direct3D	Direct3D
Docker	Docker
doParallel	R library for parallel execution
Elasticsearch	Distributed RESTful search engine built for the cloud
ERP	Enterprise resource planning system
ExtruOnt	ExtruOnt
EYE	Data storage and analysis system
fastcluster	R library for clustering
Flask	Flask
Flatform	Big data platform
foreach	R library for parallel execution
freqdom	R package freqdom
Fuseki	Apache Jena Fuseki (SPARQL server)
GADPL	generic anomaly detection for production lines
GAI	Google AI Platform
GPyOpt	Python open-source library for Bayesian Optimization
Hadoop	Framework for processing of large data sets
HealthMon	HealthMon
Hermit	Hermit
Imblearn	Python imbalanced-learn API
InfluxDB	Database
iSTEP	Integrated self-tuning engine for predictive maintenance
JavaScript	Programming language
Jupyter	Open-source web application for Python language to create and share documents
Kafka	Streaming platform
KafkaStreams	Kafka Streams
Keras	Neural Network library for Python
Kibana	Browser-based analytics and search dashboard for Elasticsearch
Knime	Data analytics, reporting, and integration platform
kohonen	R package Kohonen self-organizing maps (KSOM)
MATLAB	Programming platform
MES	Manufacturing execution systems
MLlib	Machine learning library
MongoDB	Database
MSSQL	Microsoft SQL
MUVTIME	Desktop application designed to assist in the process of multivariate time series data visual analysis
MySQL	MySQL
Neo4j	NoSQL graph database
NiFi	System to process and distribute data
NodeJS	NodeJS
OpenCV	Open-Source Computer Vision Library
OWL	OWL
Pallet	Pallet
Pandas	Pandas
Parquet	Machine-readable columnar storage format available in the Spark+Hadoop ecosystem
PlanningVis	Visual analytics system
Protege	Protégé
PyOD	Python toolbox
Python	Programming language
PyTorch	PyTorch
PyWavelets	PyWavelets
QlikSense	QlikSense
QlikView	QlikView
R	Programming language
RAMI4.0	Reference architecture model
RDFox	RDFox
RPropMLP	Knime Node
rpud	R library for the dissimilarity matrix calculation
Ruptures	Python library for offline change point detection
SCADA	Supervisory control and data acquisition
SemML	SemML
SKLEARN	Scikit-learn: Machine Learning in Python
Spark	Unified analytics engine
SPARQL	SPARQL
SPHM	Smart prognostics and health management
SQL	Query language for relational databases
SSDT	SQL Server Data Tools
SSIS	SQL Server Integration Services
Stardog	Stardog
Storm	Real-time computation system
SWRL	Semantic Web Rule Language
t-SNE	T-distributed stochastic neighbor embedding
Tensorflow	Machine learning platform
Theano	Python library for mathematical expressions
ThunderML	Machine learning toolkit
UPTIME	Unified predictive maintenance platform
Virtuoso	Virtuoso
Weka	Graphical user interface for machine learning
XGBoost	R package XGBoost
Zeppelin	Web-based notebook that enables data-driven, interactive data analytics and collaborative documents
